# Data on histological characteristics, survival patterns and determinants of mortality among colorectal, esophageal and prostate cancer patients in Ethiopia

**DOI:** 10.1016/j.dib.2021.107279

**Published:** 2021-08-12

**Authors:** Hamid Yimam Hassen, Mohammed Ahmed Teka, Jemal Beksisa, Jilcha Diribi Feyisa

**Affiliations:** a Department of Public Health, Faculty of Medicine and Health Sciences, Mizan Tepi University, Mizan Teferi, Ethiopia; b Department of Family Medicine and Population Health, University of Antwerp, Antwerp, Belgium; c School of Public Health, Saint Paul's Hospital Millennium Medical College, Addis Ababa, Ethiopia; d Department of Oncology, Saint Paul's Hospital Millennium Medical College, Addis Ababa, Ethiopia

**Keywords:** Histological characteristics, Survival, Determinant, Colorectal, Esophageal, Prostate, Cancer, Ethiopia

## Abstract

This article describes data collected retrospectively on a cohort of esophageal, colorectal and prostate cancer patients registered in the patient log book of Tikur Anbessa Specialized Hospital, Ethiopia, from January 1, 2012 to December 31, 2017. The key variables studied include histological characteristics of each type of cancer, clinical and TNM stages, baseline laboratory results (Carcinoembryonic antigen (CEA) for colorectal cancer, Prostate-Specific Antigen (PSA) for prostate cancer, hemoglobin level, etc.), clinical characteristics including sign and symptoms, family history of cancer, diagnostic and treatment modalities a patient received for each type of cancer. The event status (death) was also collected using death certificates (whenever available) and supplemented by telephone interviews with the patient or attendant. Furthermore, lifestyle characteristics of patients including tobacco use, alcohol consumption, khat (‘*Catha edulis*’) chewing, etc. and socioeconomic characteristics including age, sex, region of residence, marital status, and educational level were also collected. The aim that led to conduct the study that generated these data was to describe clinical presentation, histological characteristics, survival pattern, and to identify determinants of mortality among cancer patients in Ethiopia. Thus, independent survival analyzes were performed using Kaplan-Meier estimates and life table analysis. Furthermore, Cox's proportional hazards regression was developed to investigate the survival pattern and determinants of cancer specific mortality among colorectal, esophageal and prostate cancer patients.

## Specifications Table

**Table:** 

Subject	Health and medical sciences
Specific subject area	Cancer characteristics and survival pattern
Type of data	Primary data (Tables)
How data were acquired	Medical chart review using abstraction form and telephone interviews
Data format	• Cleaned raw data • Analyzed life table analysis accompanying this paper
Parameters for data collection	Medical charts of patients diagnosed with colorectal, esophageal or prostate cancer
Description of data collection	Data was collected using a data abstraction form and telephone interview with the patient and/or the attendant. The data abstraction form is available in the supplementary material (Annex 1)
Data source location	Institution: Tikur Anbessa Specialized Hospital City: Addis Ababa Country: Ethiopia
Data accessibility	Dataset is hosted on a public repository [1] Repository name: Mendeley Data Data identification number: https://doi.org/10.17632/vvzw3wkx93.3 Direct URL to data: https://data.mendeley.com/datasets/vvzw3wkx93/3
Related research article [2], [3], [4]	1. Teka MA, Yesuf A, Hussien FM, Hassen HY. Histological characteristics, survival pattern and prognostic determinants among colorectal cancer patients in Ethiopia: A retrospective cohort study. Heliyon. 2021;7(2):e06366. doi: https://doi.org/10.1016/j.heliyon.2021.e06366. 2. Hassen HY, Teka MA, Addisse A. Survival Status of Esophageal Cancer Patients and its Determinants in Ethiopia: A Facility Based Retrospective Cohort Study. Front Oncol. 2021 Feb 15;10:594342. https://doi.org/10.3389/fonc.2020.594342. PMID: 33659206; PMCID: PMC7917207. 3. Beksisa J, Getinet T, Tanie S, Diribi J, Hassen HY (2020) Survival and prognostic determinants of prostate cancer patients in Tikur Anbessa Specialized Hospital, Addis Ababa, Ethiopia: A retrospective cohort study. PLOS ONE 15(3): e0229854. https://doi.org/10.1371/journal.pone.0229854.

## Value of the Data

• These data fill a major gap in cancer related data and provide an overview of patients’ sociodemographic characteristics, clinical presentation, histology types and survival pattern in Ethiopia.
• The data can be utilized by researchers to investigate patterns of clinical presentation, treatment options, survival pattern and predictors of mortality as well as by clinicians to have an overall insight of their patients survival and prognosis.
• Findings of these data could guide public health professionals and clinicians working on cancer prevention and treatment to establish a screening program and early initiation of treatment to improve progression-free survival and quality of life.
• Researchers working on cancer prevention and treatment can explore diagnostic and treatment patterns, and identify further determinants of survival using these data.

## 1 Data Description

Due to a change in lifestyle [5,6], the cancer burden is increasing in low- and middle-income countries (LMICs) [7,8] including Ethiopia [9]. Investigating survival patterns of cancer patients is crucial, however, due to lack of patient outcome in routine medical records, survival estimation is not straightforward. Furthermore, the available medical records are not organized in a way that can inform clinical or public health practice. In response, we extracted all patient characteristics from medical charts and supplemented with a telephone interview for event status and other incomplete lifestyle factors. As a result, we have three datasets for esophageal, colorectal and prostate cancer which include information on sociodemographic characteristics, signs and symptoms, imaging and histological reports, and stages of cancer. Furthermore, the datasets also contain the type of diagnostic work-up and treatment options a patient received (surgery, chemotherapy, and/or radiotherapy). In addition, the event status (whether the patient died or not) and the date of the event are also available. The datasets are publicly available in Mendeley Data (https://data.mendeley.com/datasets/vvzw3wkx93/3), a secure cloud-based repository to store data, which is easy to share, access and cite from anywhere. Three datasets are stored as a .CSV file in separate folders for each cancer site. To comply with the ethical standards, identifiers such as name, medical registration number, physical address and telephone numbers are excluded from the datasets. Details of the analyzed data including sociodemographic characteristics, survival curves, and determinants of mortality among colorectal, esophageal and prostate cancer patients are available elsewhere [2], [3], [4]. Life table analyzes were computed for each cancer and the results are presented in this paper. Table 1 shows the results of life table analysis among colorectal cancer patients in TASH, Ethiopia. Likewise, Tables 2 and 3, respectively show the results of life table analysis of esophageal and prostate cancer patients.

**Table 1:** Life table analysis of patients with colorectal cancer (161) in TASH, Addis Ababa, Ethiopia, 2010–2017.

Interval (months)	Total at risk at beginning	Deaths	Lost to FU	Survival	Std. Error	(95% CI)
(0, 5)	161	9	11	0.942	0.019	(0.892, 0.969)
(5, 10)	141	17	12	0.824	0.032	(0.752, 0.876)
(10, 15)	112	25	14	0.627	0.042	(0.540, 0.703)
(15, 20)	73	14	10	0.498	0.045	(0.407, 0.583)
(20, 25)	49	3	8	0.465	0.046	(0.373, 0.552)
(25, 30)	38	3	6	0.425	0.048	(0.331, 0.516)
(30, 35)	29	1	2	0.410	0.048	(0.315, 0.502)
(35, 40)	26	1	6	0.392	0.049	(0.296, 0.487)
(40, 45)	19	1	4	0.369	0.052	(0.269, 0.469)
(45, 50)	14	1	2	0.341	0.055	(0.236, 0.448)
(50, 55)	11	0	5	0.341	0.055	(0.236, 0.448)
(55, 60)	6	0	6	0.341	0.055	(0.236, 0.448)

FU: Follow-up; 95%CI: 95% Confidence Interval; TASH: Tikur Anbessa Specialized Hospital.

**Table 2:** Life table analysis of patients with esophageal cancer (*n*=349) in TASH, Addis Ababa, Ethiopia, 2010–2017.

Interval (months)	Total at risk at beginning	Deaths	Lost to FU	Survival	Std. Error	(95% CI)
(0, 5)	349	223	0	0.361	0.026	(0.311, 0.4113)
(5, 10)	126	73	0	0.152	0.019	(0.116, 0.192)
(10, 15)	53	27	0	0.075	0.014	(0.050, 0.105)
(15, 20)	26	8	0	0.052	0.012	(0.032, 0.078)
(20, 25)	18	5	0	0.037	0.010	(0.021, 0.061)
(25, 30)	13	5	0	0.023	0.008	(0.011, 0.043)
(30, 35)	8	5	0	0.009	0.005	(0.002, 0.023)
(40, 45)	3	1	0	0.006	0.004	(0.001, 0.019)
(70, 75)	2	1	0	0.003	0.003	(0.0003, 0.015)
(75, 80)	1	1	0	0.000	.	-

FU: Follow-up; 95%CI: 95% Confidence Interval; TASH: Tikur Anbessa Specialized Hospital.

**Table 3:** Life table analysis of patients with prostate cancer (*n*=137) in TASH, Addis Ababa, Ethiopia, 2010–2017.

Interval (months)	Total at risk at beginning	Deaths	Lost to FU	Survival	Std. Error	(95% CI)
(0, 5)	137	34	0	0.752	0.037	(0.670, 0.816)
(5, 10)	103	23	0	0.584	0.042	(0.497, 0.661)
(10, 15)	80	28	0	0.380	0.042	(0.299, 0.460)
(15, 20)	52	10	0	0.307	0.039	(0.232, 0.385)
(20, 25)	42	10	0	0.234	0.036	(0.167, 0.307)
(25, 30)	32	13	0	0.139	0.030	(0.087, 0.202)
(30, 35)	19	2	0	0.124	0.028	(0.076, 0.185)
(35, 40)	17	2	0	0.110	0.027	(0.064, 0.168)
(40, 45)	15	5	0	0.073	0.022	(0.037, 0.124)
(45, 50)	10	1	0	0.066	0.021	(0.032, 0.115)
(50, 55)	9	3	0	0.044	0.018	(0.018, 0.088)
(55, 60)	6	6	0	0.000	.	.

FU: Follow-up; 95%CI: 95% Confidence Interval; TASH: Tikur Anbessa Specialized Hospital.

## 2 Experimental Design, Materials and Methods

### 2.1 Study setting and period

The data was collected from the patient log book of Tikur Anbessa Specialized Hospital (TASH) cancer treatment center. TASH is the largest tertiary level referral hospital in Ethiopia equipped with cancer diagnostic and treatment facilities. Before 2016, Tikur Anbessa was the only hospital with cancer treatment including chemotherapy and radiotherapy. Cancer cases from all parts of the country were being referred to this hospital for available care and management. The Addis Ababa Population-Based Cancer Registry (AAPBCR) was established in 2011 under the TASH oncology center, to facilitate documentation of cancer cases for research and decision making in public health policy. The registry employs active data collection methods organized by the director, supervisors, data collectors, data clerk, and focal persons at outpatient clinics and inpatient wards. The main sources of cases for the registry are general hospitals, pathology centers, and higher diagnostic clinics. Using the registry and patient log book as a source, we collected data retrospectively among all registered esophageal, colorectal and prostate cancer patients who were diagnosed or referred from January 1, 2010 to May 31, 2017. The time of diagnosis with the respective cancer was taken as the starting point of the study, while the date of death, loss to follow-up, last contact or the end of follow-up time was the end point.

### 2.2 Study design, participants and eligibility criteria

A retrospective follow up study was conducted among patients who visited TASH for cancer treatment. All clinically and pathologically confirmed esophageal, colorectal and prostate cancer cases confirmed by oncologists were included. Patient charts were excluded when both the histopathology and cancer stage report information were not available. Furthermore, due to the subjective variation across physicians, suspected cancer cases were excluded from this study. Out of 367 (esophageal), 174 (colorectal), and 171 (prostate) charts retrieved, 18 (4.9%), 13 (7.5%) and 34 (19.9%), respectively were excluded due to unavailability of neither histopathology nor cancer stage report. Consequently, all relevant patient characteristics from 349 esophageal, 161 colorectal and 137 prostate cancer patient charts were extracted and included in the dataset as well as the analysis.

### 2.3 Data collection tools, procedure and analysis

To put the patient characteristics in an organized format, we designed a structured medical record data abstraction form. We reviewed literature and consulted experts (oncologists, epidemiologists, etc.) to identify important variables for our study. Moreover, we also took into account the availability of information on patient medical charts and feasibility to collect it via a telephone interview. We followed a series of steps to extract the necessary patient information. First, we prepared a list of all cases of esophageal, colorectal, and prostate cancer patients using records from the log book of cancer patients. Second, we retrieved detailed individual medical charts from the cancer treatment center using the medical registration number as a unique identifier. Third, using the structured data abstraction form, data collectors (oncology nurses) extracted all relevant patient information from medical charts and patient log books. Data collectors reviewed sociodemographic characteristics (such as age, sex, marital status, level of education, region of residence, etc.), lifestyle factors (such as tobacco use, alcohol consumption, khat chewing, etc.) and family history of cancer, baseline and follow-up patient characteristics including sign and symptoms, laboratory (hemoglobin level, blood count, organ function tests, etc.) and imaging results (chest x-ray, ultrasound, etc.), pathology report (such as cancer stage, histology type, metastasis, etc.), and care and treatment a patient received (such as surgery, chemotherapy, radiotherapy, etc.). Fourth, a telephone interview was conducted to supplement the missing information. For a few patients who experienced the event (death) at the healthcare facility, the death certificate was identified from the TASH cancer center and relevant information was taken from it. When the death certificate was not available, which is the case for majorities, we carried out a telephone interview with patients or their attendants. Besides the event status, the sociodemographic characteristics and lifestyle factors that were not available in the patients’ medical chart or registry were collected during the telephone interview.

In this study, an event for each cancer was defined as the death of a patient due to each respective cancer type. On the other hand, patients were censored to the last follow-up date when either of the following criteria fulfilled: (i) lost to follow-up before the event, (ii) incomplete information on the date of death, (iii) those who died due to other known causes unrelated to each respective cancer, and (iv) who do not have registered telephone numbers and their status was unknown. Likewise, patients who survived until the last follow-up date were censored to May 31, 2017. Nurses who were working at the cancer treatment center performed the data extraction and facilitation of telephone interviews. To improve the data quality, a two-days training was given for data collectors on the aim, materials and methods, and procedure of data collection.

Data from the abstraction form were checked for completeness and entered into Epi Info version 7.1 (Center for Disease Control and Prevention, Atlanta). Then, electronic data was exported to R software for further processing and analysis. We performed descriptive statistical analysis and presented categorical variables in absolute and relative frequency. Whereas for numerical variables, we checked the normality employing histograms and q-q plots and we summarized using either mean with standard deviation or median with interquartile range depending on the symmetry. Independent survival analyzes were performed for each cancer using Kaplan-Meier estimate along with a log-rank test. Furthermore, Cox's proportional hazards regressions were performed to investigate the survival pattern and determinants of cancer-specific mortality among colorectal, esophageal and prostate cancer patients. Detailed results including Kaplan-Meier survival curves are available elsewhere [2], [3], [4]. We also performed a life table analysis for each cancer within five months of interval and the results are summarized in this article. All the analyzes were performed using the free statistical software R version 3.6.1 [10].

## Ethics Statement

The institutional review board of Saint Paul's Hospital Millennium Medical College and Addis Ababa University, College of Health Sciences provided ethical clearance for this study (Ref. No: PM23/92). Informed consent was also obtained from patients or caretakers before starting the telephone interview. Patients' data was stored anonymously and at each step of data collection and processing confidentiality was assured. The study was in compliance with the principles of the declaration of Helsinki.

## CRediT Author Statement

**Hamid Yimam Hassen:** Conceptualization, Methodology, Software, Original draft preparation, Visualization, Data curation, Investigation, supervision, Writing - Reviewing and Editing; **Mohammed Ahmed Teka** and **Jemal Beksisa:** Data curation, Visualization, Investigation, Data collection, Software, Validation, Writing - reviewing and editing; **Jilcha Diribi Feyisa:** Data curation, Visualization, Data Collection, Writing - reviewing & editing.

## Declaration of Competing Interest

The authors declare that they have no known competing financial interests or personal relationships which have or could be perceived to have influenced the work reported in this article.

## Appendix Supplementary materials

Image, application 1
