# Supplementary material for: Data on histological characteristics, survival patterns and determinants of mortality among colorectal, esophageal and prostate cancer patients in Ethiopia
Source: Data Brief. 2021 Aug 12;38:107279. doi: 10.1016/j.dib.2021.107279 (PMC8367784; doi:10.1016/j.dib.2021.107279)
Supplement: Supplementary file 1 [file mmc1.pdf]

Data abstraction form to collect data on histological characteristics, survival patterns and related factors of colorectal, esophageal and prostate cancer in Tikur Anbessa Specialized Hospital, Ethiopia

Checked for completeness: **Yes** ☐ **No** ☐

Name of data collector: ______________________________

Date: ______/_______/_____________ **(dd/mm/yyyy)**

Signature:_________________

Data abstraction form

I. Identification and socioeconomic characteristics

Information	Options	Remark
Unique code	_______________________	
Medical registration number	_____________________	
Date of data extraction	___/___/______(dd/mm/yyyy)	
Date of diagnosis	___/___/______(dd/mm/yyyy)	
Last date of FU	___/___/______(dd/mm/yyyy)	
Age at diagnosis (years)	____________	
Sex (*only for esophageal and colorectal cancer*)	1. Male<br>2. Female	
Region	1. Addis Ababa<br>2. Afar<br>3. Amhara<br>4. Benishangul Gumuz<br>5. Dire Dawa<br>6. Gambella<br>7. Harari<br>8. Oromia<br>9. SNNP<br>10. Somale<br>11. Tigray	
Marital status	1. Married<br>2. Single<br>3. Widowed<br>4. Divorced	
Educational status (*completed level*)	1. No formal education<br>2. Primary level<br>3. Secondary level<br>4. Higher education	

II. Lifestyle risk factors and family history of cancer

Characteristics	Category	Remark
Alcohol consumption	1. Yes<br>2. No	
Tobacco use (any type)	1. Yes<br>2. No	
Chew Khat	1. Yes<br>2. No	
Family history of cancer	1. Yes<br>2. No	
Chronic comorbidity	1. No<br>2. Yes (specify)_______	

Any other additional notes

Esophageal cancer

I. Clinical, laboratory and histopathology report

Unique code	____________________	
Symptoms	Yes (duration in days)	No
1. Difficulty of swallowing	_______	
2. Pain during swallowing	_______	
3. Weight loss	_______	
4. Vomiting	_______	
5. Heart burn	_______	
6. Cough	_______	
7. Chest pain	________	
8. Others (specify)___________	________	
Laboratory	Yes	No
1. Hemoglobin (*if yes, put the result*)	_____________ (g/dl)	
2. Complete blood count (CBC)		
3. Alanine transaminase (ALT)		
4. Aspartate transaminase (AST)		
5. Alkaline phosphatase (ALP)		
6. Others (specify) ___________ ___________		
Diagnostic workup	Yes	No
1. Endoscopy		
2. Barium swallow		
3. CT-scan		
4. Biopsy		
5. Chest X-ray		
6. Ultrasound		
7. Others (specify)		

II. Histopathology report and clinical characteristics

**Histopathology characteristics**	**Options**	**Remark**
1. Histology report available	1. Yes<br>2. No	 Skip to Q4
2. Histology type	1. Adenocarcinoma<br>2. Squamous cell carcinoma<br>3. Other (specify)_______<br>4. Not specified	
3. Histological grade	1. Well differentiated<br>2. Moderately differentiated<br>3. Poorly differentiated<br>4. Undifferentiated<br>5. Not specified	
4. Location of lesion	1. Upper-third<br>2. Middle-third<br>3. Lower-third<br>6. Unspecified	
5. Adjacent organ involvement	1. Yes<br>2. No	
6. Adjacent organ involvement site (select all that apply)	1. Trachea<br>2. Aorta	

	3. Pleura<br>4. Pericardium<br>5. Diaphragm<br>6. Vertebral body<br>7. Other (Specify) _________<br>8. Not known	
7. Distant metastasis	1. Yes<br>2. No	 Skip to Q9
8. Site of distant metastasis (select all that apply)	1. Liver<br>2. Lung<br>3. Bone<br>4. Other (specify)<br>5. Not specified	
9. TNM stage at diagnosis	______________	

III. Management and treatment options

Management and treatment options	Options	Remark
1. Gastrostomy tube inserted for feeding	1. Yes<br>2. No	
2. Surgery (esophagectomy) done	1. Yes<br>2. No<br>3. Unknown	 Skip to Q5<br>Skip to Q5
3. Type of surgery	1. Trans-Hital<br>2. Trans-Thoracic<br>3. Not specified	
4. Chemotherapy received	1. Yes<br>2. No<br>3. Not specified	 Skip to Q7<br>Skip to Q7
5. Type of chemotherapy administered	1. Adjuvant<br>2. Radical<br>3. Palliative<br>4. Not specified	
6. Number of cycles of chemotherapy a patient received	______________	
7. Radiotherapy received	1. Yes<br>2. No<br>3. Not specified	 Skip to Q9<br>Skip to Q9
8. Type of Radiotherapy administered	1. Adjuvant<br>2. Radical<br>3. Palliative<br>4. Not specified	
9. Duration of stay at hospital (months)	________	

Event status	1. Dead<br>2. Alive<br>3. Unknown	
If dead, specify date of death	____/_____/_________ (dd/mm/yyyy)	

Colorectal cancer

I. Clinical, laboratory and histopathology report

Unique code	______________________	
Symptoms	Yes (duration in days)	No
1. Diarrhea	_________	
2. Constipation	_________	
3. Blood in stool	_________	
4. Abdominal pain	_________	
5. Weight loss	_________	
6. Fatigue	_________	
7. Others (specify)__________	_________	
__________	_________	
Laboratory	Yes	No
1. Hemoglobin (*if yes, put the result*)	_____________ (g/dl)	
2. Carcinoembryonic antigen (CEA) (*if yes, put the result*)	____________ (ng/ml)	
3. Complete blood count (CBC)		
4. Alanine transaminase (ALT)		
5. Aspartate transaminase (AST)		
6. Alkaline phosphatase (ALP)		
7. Others (specify) __________ __________		
Diagnostic workup	Yes	No
1. Colonoscopy		
2. CT-scan		
3. Biopsy		
4. MRI		
5. Ultrasound		
6. Chest X-ray		
7. Other		

II. Histopathology report and clinical characteristics

**Histopathology characteristics**	**Options**	**Remark**
1. Histology report available	1. Yes<br>2. No	 Skip to Q4
2. Histology type	1. Adenocarcinoma<br>2. Squamous cell carcinoma<br>3. Mucinous carcinoma<br>4. Singent-ring cell carcinoma<br>5. Other (specify) _________<br>6. Not specified	
3. Histological grade	1. Well differentiated<br>2. Moderately differentiated<br>3. Poorly differentiated<br>4. Anaplastic<br>5. Unknown<br>6. Not specified	
4. Tumor location	1. Colon<br>2. Recto Sigmoid Junction<br>3. Rectum<br>7. Anorectal	

5. TNM stage	______________	
6. Clinical stage	1. Localized<br>2. Locally advanced<br>3. Metastasis<br>8. Unspecified	
7. Regional lymph nodes Involved	1. Yes<br>2. No	
8. Metastasis	1. Yes<br>2. No	 Skip to Q10
9. Site of distant metastasis (select all that apply)	1. Liver<br>2. Lung<br>3. Peritoneum<br>4. Other (specify)___________<br>5. Not specified	
10. Vascular invasion	1. Yes<br>2. No<br>3. Unspecified	
11. Residual tumor identified	1. Yes<br>2. No<br>3. Unspecified	

III. Management and treatment options

**Management and treatment options**	**Options**	**Remark**
1. Surgery done	1. Yes<br>2. No<br>3. Unknown	
2. Chemotherapy received	1. Yes<br>2. No<br>3. Not specified	 Skip to Q4<br>Skip to Q4
3. Number of cycles of chemotherapy a patient received	______________	
4. Radiotherapy received	1. Yes<br>2. No<br>3. Not specified	 Skip to Q6<br>Skip to Q6
5. Number of cycles of radiotherapy a patient received	______________	
6. Duration of stay at hospital (months)	______________	

Event status	1. Dead<br>2. Alive<br>3. Unknown	
If dead, specify date of death	____/_____/_________ (dd/mm/yyyy)	

Prostate cancer

I. Clinical, laboratory and histopathology report

Unique code	____________________	
Symptoms	Yes (duration in days)	No
1. Frequent urination	________	
2. Blood in the urine	________	
3. Erectile dysfunction	________	
4. Pain/burning during urination	________	
5. Bone pain	________	
6. Others (specify)__________	________	
Laboratory	Yes (result)	No
1. Prostate-specific antigen (PSA) (*if yes, put the result*)	_________ ng/mL	
2. Hemoglobin (*if yes, put the result*)	_________ (g/dl)	
3. Complete blood count (CBC)		
4. Urine analysis		
5. Serum creatinine		
6. Others (specify) __________ __________		
Diagnostic workup	Yes	No
1. Ultrasound		
2. Biopsy		
3. CT-scan		
4. MRI		
5. Others (specify) ________________		

II. Histopathology report and clinical characteristics

**Histopathology characteristics**	**Options**	**Remark**
1. TNM stage	______________	
2. Gleason score	______________	
3. Histology report available	1. Yes<br>2. No	 Skip to Q6
4. Histology type	1. Adenocarcinoma<br>2. Sarcoma<br>3. Other (specify)_______<br>4. Not specified	
5. Histological grade	1. Well differentiated<br>2. Moderately differentiated<br>3. Poorly differentiated<br>4. Undifferentiated<br>5. Not specified	
6. Lymph node involvement	1. Yes<br>2. No	
7. Bone metastasis	1. Yes<br>2. No	
8. Distant metastasis	1. Yes<br>2. No	 Skip to sec. III
9. Distant metastasis site (*select all that apply*)	1. Liver<br>2. Lung<br>3. Other (Specify) _________<br>4. Not specified	

III. Management and treatment options

Management and treatment options	Options	Remark
1. Surgery (orchiectomy) done	1. Yes<br>2. No<br>3. Unknown	 Skip to Q3<br>Skip to Q3
2. Type of orchiectomy	1. Simple<br>2. Subcapsular<br>3. Inguinal<br>4. Not specified	
3. Androgen deprivation therapy	1. Yes<br>2. No<br>3. Not specified	
4. Chemotherapy received	1. Yes<br>2. No<br>3. Not specified	 Skip to Q6<br>Skip to Q6
5. Number of cycles of chemotherapy a patient received	______________	
6. Radiotherapy received	1. Yes<br>2. No<br>3. Not specified	 Skip to Q8<br>Skip to Q8
7. Number of cycles of chemotherapy a patient received	______________	
8. Duration of stay at hospital (months)	______________	

Event status	1. Dead<br>2. Alive<br>3. Unknown	
If dead, specify date of death	____/_____/_________ (dd/mm/yyyy)	
